# Predicting body composition using foot-to-foot bioelectrical impedance analysis in healthy Asian individuals

**DOI:** 10.1186/s12937-015-0041-0

**Published:** 2015-05-19

**Authors:** Chun-Shien Wu, Yu-Yawn Chen, Chih-Lin Chuang, Li-Ming Chiang, Gregory B. Dwyer, Ying-Lin Hsu, Ai-Chun Huang, Chung-Liang Lai, Kuen-Chang Hsieh

**Affiliations:** 1 Center for General Education, I-Shou University, No.1 Sec. 1, Syuecheng Rd., Dashu Dist, Kaoshiung City, 84001 Taiwan; 2 Department of Physical Education, National Taiwan Sport University, No. 16, Sec. 1, Shuang-Shih Road, Taichung City, 404 Taiwan; 3 Department of Radiology, Jen-Ai Hospital, No. 483, Don Long Road, Dali Dist, Taichung City, 412 Taiwan; 4 Department of Hotel, Restaurant, and Tourism Management, College of Business and Management, East Stroudsburg University of Pennsylvania, 200 Prospect St., East Stroudsburg, PA 18301 USA; 5 Department of Exercise Science, College of Health Science, East Stroudsburg University of Pennsylvania, 200 Prospect St., East Stroudsburg, PA 18301 USA; 6 Department of Applied Math, National Chung Hsing University, 250 Kuo Kuang Rd., Taichung City, 402 Taiwan; 7 Department of Leisure, Recreation and Tourism Management, Tzu-Hui Institute of Technology, No. 367, Sanmin Rd., Nanjhou Hsian, Pingtung County 926 Taiwan; 8 Department of Physical Medicine and Rehabilitation, Taichung Hospital, Ministry of Health and Welfare, No. 199, Sec. 1, San-Min Road, Taichung City, 403 Taiwan; 9 Office of Physical Education and Sport, National Chung Hsing University, 250 Kuo Kuang Rd., Taichung City, 402 Taiwan; 10 Research Center, Charder Electronic Co., LTD, No. 103, Guozhong Rd., Dali Dist., Taichung City, 412 Taiwan

**Keywords:** Dual-energy X-ray absorptiometry, Foot-to-foot, Cross-validation, Fat-free mass

## Abstract

**Background:**

The objectives of this study were to develop a regression model for predicting fat-free mass (FFM) in a population of healthy Taiwanese individuals using standing foot-to-foot bioelectrical impedance analysis (BIA) and to test the model’s performance in predicting FFM with different body fat percentages (BF%).

**Methods:**

We used dual-energy X-ray absorptiometry (DXA) to measure the FFM of 554 healthy Asian subjects (age, 16–75 y; body mass index, 15.8–43.1 kg/m^2^). We also evaluated the validity of the developed multivariate model using a double cross-validation technique and assessed the accuracy of the model in an all-subjects sample and subgroup samples with different body fat levels.

**Results:**

Predictors in the all-subjects multivariate model included height^2^/impedance, weight, year, and sex (FFM = 13.055 + 0.204 weight + 0.394 height^2^/Impedance – 0.136 age + 8.125 sex (sex: Female = 0, Male = 1), r^2^ = 0.92, standard error of the estimate = 3.17 kg). The correlation coefficients between predictive FFM by BIA (FFM_BIA_) and DXA-measured FFM (FFM_DXA_) in female subjects with a total-subjects BF%_DXA_ of <20 %, 20 %–30 %, 30 %–40 % and >40 % were r = 0.87, 0.90, 0.91, 0.89, and 0.94, respectively, with bias ± 2SD of 0.0 ± 3.0 kg, −2.6 ± 1.7 kg, −1.5 ± 2.8 kg, 0.5 ± 2.7 kg, and 2.0 ± 2.9 kg, respectively. The correlation coefficients between FFM_BIA_ and FFM_DXA_ in male subjects with a total-subjects BF%_DXA_ of <10 %, 10 %–20 %, 20 %–30 %, and >30 % were r = 0.89, 0.89, 0.90, 0.93, and 0.91, respectively, with bias ± 2SD of 0.0 ± 3.2 kg, −2.3 ± 2.5 kg, −0.5 ± 3.2 kg, 0.4 ± 3.1 kg, and 2.1 ± 3.2 kg, respectively.

**Conclusions:**

The standing foot-to-foot BIA method developed in this study can accurately predict FFM in healthy Asian individuals with different levels of body fat.

## Background

In recent years, bioelectrical impedance analysis (BIA) has undergone major changes. The traditional electrodes that are pasted on with gel have been replaced with reusable stainless steel contact electrodes [1, 2], and the measuring position has been changed from supine to standing. In standing foot-to-foot BIA, impedance is measured through the electronic pathway of the lower extremities [3]. This technique is widely used to assess the whole-body composition [4–6].

Although many fat-free mass (FFM) predictive models based on BIA performed in the supine position have been validated, most of these models have been developed for Caucasian, African-American, Hispanic, and Native American populations [7–9]. Insufficient research has been performed to develop FFM predictive models based on BIA in Asian populations. Because different ethnic groups may exhibit different body composition characteristics, predictive models should be developed for specific populations to prevent ethnic bias and facilitate accurate estimates of FFM [10, 11].

Standing foot-to-foot BIA may be a convenient and safe method for assessing body composition. In the existing research, however, there is inconsistent support of the validity of BIA in estimating FFM in the general population [4, 6, 8, 12–17], BIA cannot accurately estimate FFM in obese populations [18–20], BIA tends to underestimate the body fat percentage (BF%) and overestimate the FFM, and body composition parameters are affected by the BF% in tested subjects.

Studies to date have only explored the regression mode for tested subjects because manufacturers retain the prediction equations of the measuring system as confidential information; therefore, knowledge of BIA devices is limited [13]. Additionally, studies to date have had relatively small sample sizes that do not represent the entire population, thereby minimizing the generalizability and external validity of the results.

## Methods

### Subjects

In total, 554 Taiwanese subjects were recruited by advertisement and volunteered to participate in this study. The subjects were asked to complete a health questionnaire that included personal background information, physical characteristics, and health status. The subjects were tested following 48 h without alcohol, 7 days without diuretic agents, and 24 h without strenuous physical activity. No urination was allowed within 30 min prior to the BIA and DXA measurements. Female participants were not tested during their menses. No subjects reported a history of endocrine, nutritional, or growth disorders; chronic illnesses such as high blood pressure, diabetes, cancer, kidney dysfunction, liver disease, or asthma; or electronic device insertion. Each subject underwent foot-to-foot BIA and DXA at the Department of Radiology of Dah Li County Jen-Ai Hospital in Taiwan. This study was approved by the institutional review board of Jen-Ai Hospital.

### Anthropometry

The subjects were weighed to the nearest 0.1 kg on a Weight-Tronix scale, (Scale Electronics Development, NY, USA). Height was measured without shoes to the nearest 0.5 cm on a stadiometer (Holtain Ltd., Crosswell, Wales, UK). Body mass index (BMI) was calculated as weight divided by height squared (kg/m^2^).

### Measurements of body composition

The subjects were asked to wear a cotton gown and remove all metallic objects from the body. Body composition was measured using a DXA system (Lunar Prodigy; GE Healthcare, Madison, WI, USA). The estimation of body composition included measurement of total body fat, fat-free soft tissue, and bone mineral content. The BF% was calculated as 100 × fat mass/(fat mass + FFM), where FFM was the summation of the fat-free soft tissue and mineral content. The DXA measurements were taken at 2:00 PM using enCORE 2003 Version 7.0 software (GE Healthcare), and the foot-to-foot impedance measurements were conducted following the DXA measurements. With the subjects in a standing position, the measurements were repeated until they were stable to within 5 ohm (usually up to three times within an interval of 30 s), and the average value of three repeated measurements was used in the calculations.

### Impedance analysis

The BIA measurements were taken using the impedance measurement device. We used an imbedded stainless steel contact plate with four electrodes, which was connected to a QuadScan 4000 (Bodystat, Douglas, UK). The QuadScan 4000 was in turn connected to stainless steel electrode plates. A body composition sensing platform (HBF-361; Omron Healthcare, Kyoto, Japan) was used as the impedance measuring base and included a stainless steel polar plate and cable. The subjects were asked to stand on the contact plate without shoes and with their feet slightly apart. A current of only 50 kHz was used to measure the impedance in the left-foot-to-right-foot pathway, expressed as Z_F-F_.

The coefficient of variation (CV) (standard deviation [SD]/mean), expressed as a percentage, of the within-day and between-day estimates of impedance in all subjects was calculated to evaluate the repeatability of the impedance measurements. After referring to the amount of time spent during the DXA measurement, the subjects lay in the supine position for 20 min and then underwent the foot-to-foot standing position impedance measurement. The within-day CV was calculated by measuring the impedance of ten subjects (five males and five females) ten times within 1 h, and the between-day CV was calculated by measuring the impedance of 10 subjects on five consecutive days.

All measurements were conducted in a well-ventilated room with controlled temperature and humidity. The impedance measurements were performed three times within 3 min in each subject immediately after the DXA measurements.

### Statistical analysis

The BF% derived by DXA (expressed as BF%_DXA_) was grouped by 10 % difference intervals for percent body fat [21]. The variables, which included age, height, weight, BMI, and impedance, are expressed as mean ± SD, and the numbers in parentheses are the minimum and maximum values. The paired-samples *t*-test was used to evaluate the differences between the two measurement methods.

The estimates of FFM derived by DXA were used as reference values (expressed as FFM_DXA_) to develop a stepwise multiple regression model by setting height squared/impedance (h^2^/Z_F-F_), weight (w), age (y), and sex (s, female: 0, male: 1) as predictive variables. The stepwise procedure included the variable with the highest correlation coefficient and the minimum standard error of estimation in the model first. The FFM model predicted by BIA was developed using a double cross-validation technique. The subjects were split into two groups, G_1_ and G_2_, based on BF%. Thus, the two groups were evenly matched for BF%. An FFM predictive model was then constructed for each group and cross-validated with each model. If the FFM predictive models were proved to be similar to each other by comparing the correlation and standard error of the estimate (SEE) [11], the two samples were combined to develop a pooled FFM predictive model [22, 23].

Furthermore, the root mean square error (RMSE) and pure error (PE) were used to test the accuracy of the FFM predictive model by BIA:

$$ \mathsf{RMSE}=\sqrt{{\displaystyle \sum}\frac{{\left({\mathrm{y}}_{\mathrm{i}}^{\hbox{'}}-{\mathrm{y}}_{\mathrm{i}}\right)}^2}{\mathrm{n}}-\mathrm{p}-1,} $$

where y’ the predicted FFM, y is the observed FFM, n is the number of subjects, and p is the number of predictor variables, and

$$ {\displaystyle \mathrm{P}\mathrm{E}=\sqrt{\sum \frac{{\left({\mathrm{y}}_{\mathrm{i}}^{\hbox{'}}-{\mathrm{y}}_{\mathrm{i}}\right)}^2}{\mathrm{n}}}}, $$

where the error of measurement estimates the magnitude of the error for a given measurement and is defined as the difference between measurements for the individual (i) (i = 1…, *n*, where n is the number of individuals).

A Bland–Altman plot [24] was performed to assess the agreement between the results from the FFM predictive model and the DXA measurements. Passing–Bablok regression describes a linear regression procedure with no special assumptions regarding the distribution of the samples and the measurement errors [25] and was used to evaluate the interchangeability of the two methods. All analyses were conducted using SPSS for Windows (Version 17.0; SPSS Inc., Chicago, IL, USA) and MedCalc (Version 9.0; MedCalc Inc., Mariakerke, Belgium). Statistical significance was set at *p* < 0.05 for all tests.

## Results

The physical characteristics of the subjects grouped by sex and BF% are shown in Table 1. A total of 554 subjects met the criteria for this study: 257 females and 297 males. The subjects ranged in age from 16 to 75 years with a mean of 32.8 ± 14.8 years. Their weight varied from 39.0 to 125.5 kg, and their BMI ranged from 15.9 to 43.1 kg/m^2^ with a mean of 24.0 ± 4.1 kg/m^2^. The BMIs for G_1_ and G_2_ were 23.9 ± 4.2 (range, 16.0–43.1) and 24.0 ± 4.1 (range, 16.2–42.9) kg/m^2^, respectively. The subjects were also divided into five different subgroups by 10 % body fat intervals (Table 1).

**Table 1:** Characteristics and impedances of subjects grouped by sex and %BF^a^

Female (*n* = 257)					
BF%_DXA_	<20 % (*n* = 22)	20 % - 30 % (*n* = 80)	30 %-40 % (*n* = 94)	>40 % (*n* = 61)	Total (*n* = 257)
Age(year)	21.6 ± 3.8	27.9 ± 12.5	37.5 ± 13.4	43.3 ± 15.4	34.7 ± 14.8
	(17, 59)	(16, 61)	(16, 67)	(18, 75)	(16,75)
Height(cm)	162.8 ± 6.8	162.1 ± 6.3	158.9 ± 6.4	158.9 ± 7.6	160.2 ± 6.9
	(151, 174)	(148, 174)	(143, 181)	(144, 178)	(143, 181)
Weight(kg)	49.3 ± 4.0	54.8 ± 6.3	58.3 ± 8.0	74.8 ± 14.5	60.2 ± 12.2
	(44, 58)	(39, 69)	(42, 84)	(53, 108)	(38, 108)
BMI (kg/m^2^)	18.9 ± 1.3	20.8 ± 2.0	23.1 ± 2.9	29.1 ± 4.5	23.5 ± 4.5
	(16, 22)	(16, 25)	(16, 31)	(22, 43)	(16, 43)
Z_F-F_ (ohm)	560.5 ± 76.0	529.2 ± 60.2	443.9 ± 58.2	488.9 ± 68.0	521.5 ± 66.1
	(446, 733)	(391, 704)	(326,595)	(362, 706)	(326, 733)
h^2^/Z_F-F_(cm^2^/ohm)	48.1 ± 7.0	50.4 ± 7.1	48.4 ± 6.3	52.6 ± 8.1	50.0 ± 7.2
	(33, 59)	(36, 69)	(33, 65)	(33, 79)	(33, 79)
Male (*n* = 297)					
BF%_DXA_	<10 % (*n* = 46)	10 % - 20 % (*n* = 96)	20 % - 30 % (*n* = 104)	>30 % (*n* = 51)	Total (*n* = 297)
Age(year)	20.3 ± 2.3	24.7 ± 10.7	35.4 ± 14.8	44.7 ± 14.6	31.2 ± 14.8
	(16, 29)	(16, 65)	(16, 71)	(21, 71)	(16,71)
Height(cm)	173.0 ± 5.7	173.5 ± 8.1	173.2 ± 6.7	172.3 ± 8.0	173.3 ± 7.3
	(160, 182)	(157, 196)	(156, 193)	(155, 200)	(155, 200)
Weight(kg)	65.0 ± 5.4	67.3 ± 8.1	76.4 ± 10.4	86.5 ± 14.5	73.4 ± 12.4
	(52, 77)	(42, 90)	(59, 122)	(66, 125)	(42, 125)
BMI (kg/m^2^)	21.7 ± 1.6	22.3 ± 1.9	25.4 ± 2.7	28.8 ± 4.1	24.4 ± 3.7
	(18, 26)	(17, 27)	(19.7, 37)	(23, 42)	(17, 42)
Z_F-F_ (ohm)	441.3 ± 38.3	451.5 ± 46.8	449.8 ± 53.9	439.9 ± 49.9	447.3 ± 48.8
	(380, 530)	(365, 685)	(334, 625)	(345, 572)	(335, 685)
h^2^/Z_F-F_(cm^2^/ohm)	68.4 ± 7.3	67.4 ± 8.4	67.8 ± 10.1	69.2 ± 10.0	68.0 ± 9.1
	(56, 84)	(36, 96)	(46, 97)	(49, 96)	(36, 97)

^a^All values are mean ± SD; minimum and maximum in parentheses, ^2^BI, bioelectrical index; h^2^/Z_F-F_, Height^2^/Impedance

The CV of the within-day estimates of impedance in ten subjects was 0.3 % to 0.8 %, and the CV of the between-day estimates of impedance in 10 subjects was 0.9 % to 1.7 %.

Table 2 summarizes the cross-validation results in which the r^2^, SE, PE, and RMSE show very similar values between the two groups. The regression lines of FFM_BIA_ against FFM_DXA_ developed using G_1_ and G_2_ data demonstrate similar trends deviated from the identical line (slope = 0.92 for G_1_ and 0.93 for G_2_).

**Table 2:** Prediction equation for G_1_ and G_2_ subjects

G_1_ subjects (*n* = 277)	
Measured FFM_DXA_	50.54 ± 11.58 kg
Prediction FFM_BIA_	12.518 + 0.215 w + 0.397 h^2^/ Z_F-F_ – 0.143 y + 7.843 S (Female = 0, Male = 1), (r^2^ = 0.92, SE = 3.23 kg, CV = 6.3 %) (1.a)
Prediction FFM	
Cross-validation using G_2_ subjects FFM	50.48 ± 10.97 kg, r = 0.96, bias ± SD = −0.06 ± 3.22 kg, PE = 3.22 kg, RMSE = 2.31 kg, LOA = −6.46 to 6.38 kg
G_2_ subjects (*n* = 277)	
Measured FFM_DXA_	49.81 ± 10.91 kg
Prediction FFM_BIA_	13.639 + 0.192 w + 0.392 h^2^/ Z_F-F_ – 0.129 y + 8.355 S (Female = 0, Male = 1), (r^2^ = 0.92, SEE = 3.13 kg, CV = 6.1 %) (1.b)
Prediction FFM	
Cross-validation using G_1_ subjects FFM	49.86 ± 10.60 kg, r = 0.96, bias ± SD = 0.05 ± 3.13 kg, PE = 3.12 kg, RMSE = 2.18 kg, LOA = −6.21 to 6.31 kg

FFM, fat free mass; Regression coefficient estimate ± SE; FFM_DXA_, DXA measurement of FFM; FFM_BIA_, BIA prediction of FFM; h^2^/Z_F-F_, height ^2^/impedance; SEE, standard error of estimate; LOA, limits of agreement

RMSE, Root mean square error= $\sqrt{{\displaystyle \sum}\frac{{\left({\mathrm{y}}_{\mathrm{i}}^{\hbox{'}}-{\mathrm{y}}_{\mathrm{i}}\right)}^2}{\mathrm{n}}-\mathrm{p}-1}$, where y’ the predicted FFM, y is the observed; n is the number of subjects, and p is the number of predictor variables; PE, Pure errors= $\sqrt{{\displaystyle \sum}\frac{{\left({\mathrm{y}}_{\mathrm{i}}^{\hbox{'}}-{\mathrm{y}}_{\mathrm{i}}\right)}^2}{\mathrm{n}}}$; r, correlation coefficient between FFM_BIA_ and FFM_DXA_

**Fig. 1:**
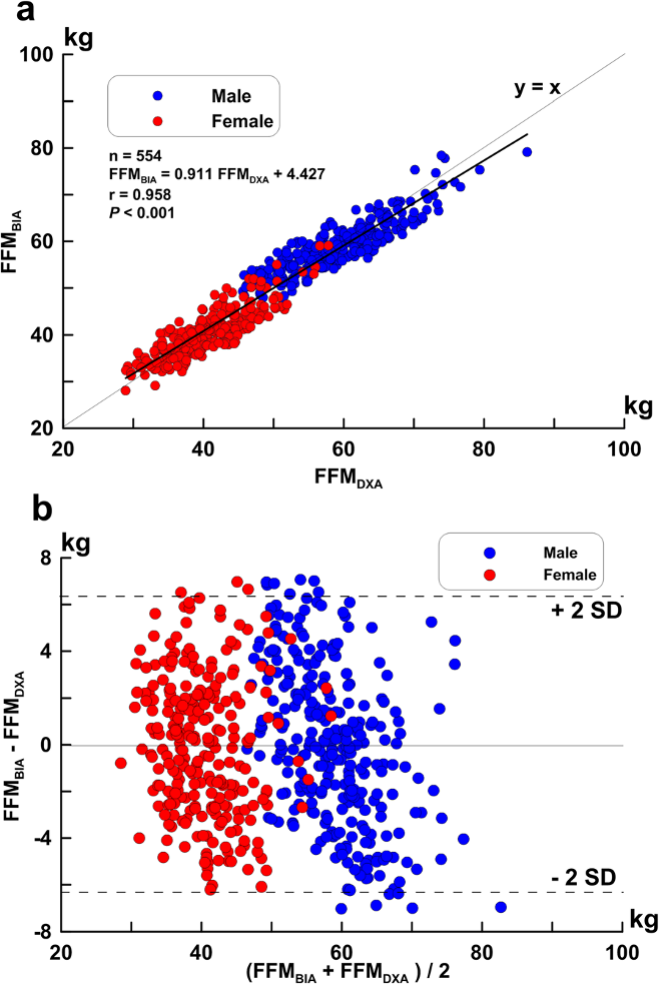
Correlations (**a**) and difference (**b**) of FFM in all subjects estimated by FFM_DXA_ and FFM_BIA_. The difference (calculated as FFM_BIA_ - FFM_DXA_ per Bland-Altman) is plotted against the mean of the measurements of FFM_DXA_ and FFM_BIA_

The results of the FFM predictive model by multiple regression analysis for all 554 subjects are shown in Table 3. Figure 1a shows the correlation for all subjects measured by FFM_DXA_ and the predictive values of FFM_BIA_. The Passing–Bablok regression analysis indicated a foot-to-foot BIA and DXA equation as follows: FFM_BIA_ = 0.911 FFM_DXA_ + 4.27 with a 95 % confidence interval (CI) of 0.80 to 1.02 for the slope and a 95 % CI of −0.74 to 9.28 for the intercept of the regression model, indicating that the foot-to-foot BIA and DXA FFM estimate methods are interchangeable (*p* > 0.10). Figure 1b shows a Bland–Altman plot of the differences between the all-subjects FFM_DXA_ and the predictive values of FFM_BIA_. For FFM, the –2SD to +2SD was −6.40 to 6.40 kg. The correlation between FFM_BIA_ – FFM_DXA_ and FFM_DXA_ can be expressed as the regression line y = −0.089 × + 4.428 (r = 0.31, *p* < 0.001).

**Table 3:** Prediction equation for FFM using all subjects

Development group (*n* =554)	
Measured FFM_DXA_	50.17 ± 11.25 kg
FFM prediction equation (FFM_BIA_)	13.055 + 0.204 w + 0.394 h^2^/ Z_F-F_ – 0.136 y + 8.125 S (Female = 0, Male = 1), (r^2^ = 0.92, SEE = 3.17 kg, CV = 6.3 %) (2)
Prediction FFM	50.17 ± 10.69 kg, PE = 3.20 kg, RMSE = 2.29 kg

FFM, fat free mass; SE, Standard error of estimate; FFM_DXA_, DXA measurement of FFM; FFM_BIA_, BIA prediction of FFM; h^2^/Z_F-F_, height^2^/impedance; SEE, standard error of estimate;

Root mean square error (RMSE) = $\sqrt{{\displaystyle \sum}\frac{{\left({\mathrm{y}}_{\mathrm{i}}^{\hbox{'}}-{\mathrm{y}}_{\mathrm{i}}\right)}^2}{\mathrm{n}}-\mathrm{p}-1}$

Pure errors (PE) = $\sqrt{{\displaystyle \sum}\frac{{\left({\mathrm{y}}_{\mathrm{i}}^{\hbox{'}}-{\mathrm{y}}_{\mathrm{i}}\right)}^2}{\mathrm{n}}}$; where y’ is the predicted FFM, y is the observed, n is the number of subjects and p is the number of predictor variables

In the developed FFM predictive model, the cumulative SEE and r^2^ by individual predictors are shown in Table 4. The determination coefficient between the predictive FFM by the single predictor h^2^/Z_F-F_ and FFM_DXA_ was r^2^ = 0.837. As the predictors sex, weight, and age were added into the model, the determination coefficients changed to r^2^ = 0.881, 0.893, and 0.922, respectively. The standard β coefficients of the predictors’ height squared/impedance, sex, weight, and age were 0.43, 0.36, 0.25, and −0.18, respectively, and the variance inflation factor (VIF) values were 4.27, 2.25, 2.49, and 1.16, respectively.

**Table 4:** Multiple stepwise regression analysis results for h^2^/Z_F-F_ measured with foot-to-foot BIA as an independent variable and FFM_DXA_ as a dependent variable

Dependent variables used in model (All subjects, *n* = 554)								Dependent variable		
h^2^/Z_F-F_	Sex	Weight	Age	Intercept	SEE(kg)	r^2^	VIF	β	r	SEE (kg)
0.84 ± 0.02^**^	-	-	-	−0.10 ± 0.96	4.54	0.84	4.27	0.43	0.92^**^	4.54
0.63 ± 0.02^**^	6.99 ± 0.49^**^	-	-	8.68 ± 1.03^**^	3.88	0.89	2.25	0.36	0.82^**^	6.52
0.52 ± 0.03^**^	7.56 ± 0.47^**^	0.14 ± 0.02^**^	-	7.09 ± 0.99^**^	3.68	0.90	2.49	0.25	0.73^**^	7.74
0.39 ± 0.02^**^	8.12 ± 0.41^**^	0.20 ± 0.02^**^	−0.14 ± 0.01^**^	13.05 ± 0.96^**^	3.16	0.92	1.16	−0.18	−0.29^**^	10.76

Regression coefficient estimate ± SE; r, variance; r^2^, determination coefficient; ^*^p < 0.05, ^**^p < 0.001; β: Standardized coefficients; VIF, variance inflation factor

Table 5 shows a comparison of the results for the mean and bias. The regression line and correlation coefficients between FFM_DXA_ and FFM_BIA_ predicted by the model for sex and BF% are shown in Table 5. The correlation coefficients of different BF%_DXA_ subgroups ranged from 0.89 to 0.94 (r = 0.89–0.94). The subgroups with the largest bias ± SD of FFM_DXA_ and FFM_BIA_ were the BF%_DXA_ > 40 % (female subgroup) and the BF%_DXA_ > 30 % (male subgroup), with bias ± SD of −2.0 ± 2.9 kg and 2.1 ± 3.2 kg, respectively. The subgroups with the smallest bias ± SD of FFM_DXA_ and FFM_BIA_ were the female subgroup and the male subgroup, with bias ± SD of 0.0 ± 3.0 kg and 0.0 ± 3.2 kg, respectively. Although the Bland–Altman plot indicates that there was a systematic error with the BIA method, this error was small. For example, the BIA method would underestimate FFM by an average of 2.2 % in an individual with an FFM of 40 kg and overestimate FFM by −1.5 % in an individual with an FFM of 60 kg.

**Table 5:** Comparison of the results of FFM_DXA and_ FFM_BIA_ for different %BF subgroups^a^

Female: BF%_DXA_	<20 % (*n* = 20)	20 % - 30 % (*n* = 80)	30 %-40 % (*n* = 94)	>40 % (*n* = 61)	Total (*n* = 257)
FFM_DXA_ (kg)	41.8 ± 3.8	41.8 ± 5.2	38.4 ± 4.7	40.9 ± 6.9	40.3 ± 5.6
	(35, 48)	(29, 52)	(29, 51)	(30, 58)	(29, 58)
FFM_BIA_ (kg)	39.2 ± 3.6	40.3 ± 4.7	38.9 ± 4.4	42.9 ± 6.7	40.3 ± 5.2
	(32, 45)	(29, 51)	(28, 51)	(31, 60)	(28, 59)
Bias ± SD^b^ (kg)	−2.6 ± 1.7	−1.5 ± 2.8	0.5 ± 2.7	2.0 ± 2.9	0.0 ± 3.0
r^,c^	0.90	0.91	0.89	0.94	0.87
*P* ^,d^	0.04^*^	0.06	0.43	0.11	0.92
Slope^,e^	0.86	0.83	0.79	0.89	0.79
Intercept^,e^	3.17	5.34	9.77	6.70	8.41
Male: BF%_DXA_	<10 % (*n* = 46)	10 % - 20 % (*n* = 96)	20 % - 30 % (*n* = 104)	>30 % (*n* = 51)	Total (*n* = 297)
FFM_DXA_(kg)	60.9 ± 5.2	58.6 ± 7.0	58.2 ± 7.9	57.9 ± 7.7	50.1 ± 11.4
	(47, 71)	(33, 76)	(46, 86)	(46, 77)	(33, 86)
FFM_BIA_(kg)	58.6 ± 3.8	58.1 ± 5.3	58.6 ± 6.7	60.0 ± 7.4	50.0 ± 10.9
	(52, 67)	(39, 74)	(48, 79)	(47, 79)	(40, 79.2)
Bias ± SD^b^(kg)	−2.3 ± 2.5	−0.5 ± 3.2	0.4 ± 3.1	2.1 ± 3.2	0.0 ± 3.2
r^,c^	0.89	0.90	0.93	0.91	0.89
*P* ^,d^	0.02^*^	0.59	0.68	0.16	0.97
Slope^e^	0.65	0.68	0.79	0.87	0.70
Intercept^e^	19.13	18.52	12.71	9.60	17.41

^a^All values are mean ± SD; minimum and maximum in parentheses

^b^The biases and standard deviations between FFM_DXA_ and FFM_BIA_ indifferent subgroups

^c^The correlation coefficients of FFM_DXA_ and FFM_BIA_ in different subgroups

^d^The results of paired *t*-tests between FFM_DXA_ and FFM_BIA_ indifferent subgroups

^e^The slopes and intercepts of the regression model of FFM_DXA_ and FFM_BIA_ indifferent subgroups

*Denotes significantly different at the .05 level

## Discussion

The present study measured FFM in 544 healthy Asian individuals using DXA (297 male, 257 female; age range, 16–75 years). We used BIA to measure the impedance at 50 kHz of the lower extremities in a standing position to develop a multivariable model for predicting FFM using DXA measurements. We also evaluated the validity of the developed multivariable model with a double cross-validation technique and assessed the accuracy of the model in an all-subjects sample and in different BF% subgroup samples. The results of the study indicated that the FFM predictive model based on BIA estimates is a valid method for assessing FFM in healthy subjects with different BF% values. The force of gravity has an effect on the fluid distribution in our body. Depending on the body position, gravity may also cause differences in blood pressures. As a result, regulation of blood volume may become challenging: standing still leads to rapid and persistent plasma volume loss of up to 7 % for a 30-min period [26]. Nunez *et al.* [1] performed foot-to-foot standing upright and supine position impedance measurements and obtained the following results. There was a high correlation between upright and supine position impedance measurements of the lower extremities. The difference in impedance measured by the two methods versus the mean impedance for the two methods was evaluated as a Bland–Altman plot (r = 0.44, *p* = 0.23, NS). The plot showed a small but systematic difference between the two methods. In a study by Rush *et al.* [27], the foot-to-hand impedance decreased by up to 9 ohm (mean, 5 ohm; 1.0 %) over 10 min of standing and increased by up to 7 ohm (mean, 3 ohm; 0.7 %) in the lying position. Based on the results of both studies, the difference in the impedance measures was caused by the changes in the effects of gravity on the different positions and body fluids. Oshima [28] reported that the average foot-to-foot impedance value would decrease by 6.8 % after 6 h of continuous measurements. Kushner *et al.* [29] also reported a −3 % to 1 % change during a 5-min standing upright position and 10-min supine position in hand-to-foot impedance measurements. In the present study, regardless of standing or lying down, only a 1 % decrease in the foot-to-foot impedance measurement occurred over a 3-min period (data not shown).

The standing foot-to-foot BIA method described herein produced inconclusive results. The present study had the following characteristics: (a) We used the same instruments in the same setting to measure the impedance by foot-to-foot BIA in the standing position and FFM by DXA in a large, single-institution Asian sample; such patients have been insufficiently studied in BIA research to date. (b) Instead of evaluating the validity of existing commercial instruments, this study aimed to develop an FFM predictive model using standing foot-to-foot BIA. (c) This study tested not only the accuracy and suitability of BIA for assessing body composition in a general population, but also its performance in subjects with different BF% values.

The present study used h^2^/Z_F-F_ and other anthropometric variables, such as weight, sex, and age, as predictive variables to develop the prediction model. We used h^2^/Z_F-F_ instead of h^2^/reactance (X_C_) or resistance (R), as adopted by other studies [23, 30], as a predictor in the regression model for the following reasons: The QuadScan 4000 produces a 50-kHz frequency and provides measured results for resistance, impedance, and reactance. Nunez *et al.* [1] proposed a standing foot-to-foot bioimpedance analysis FFM estimate model in which they used Z (impedance) as the estimate variable; this variable has also been widely used in other studies of BIA standing-position body composition estimation [31]. Furthermore, in similar research, Kotler [32] and Lukaski *et al.* [33] indicated that when using resistance or impedance estimating FFM, TBW (total body water) and TBK (total body potassium) have no significant difference. For these reasons, we used impedance as the estimated variable. When developing a body composition predictive model, the predictors should be easy to measure, accurate, reproducible, and physiologically related to the dependent variable [34, 35]; the predictors in our model meet these requirements [36, 37].

When developing a regression model, the following issues should be taken into consideration to avoid violating assumptions and to ensure that the regression analyses have sufficient power: collinearity, sample size, number of predictors, cross-validation, and SEE [38]. When additional variables were added into the FFM predictive equation using BIA and other anthropometric variables, collinearity was present if the predictive variables were highly correlated. This might affect the estimation of the regression coefficient in a predictive model, which may lead to incorrect identification of the predictive variables. The VIF analysis was conducted to identify potential problems related to collinearity. When the VIF of the predictors had exceeded ten, the collinearity was considered to be severe. In this study, the VIF was smaller than five; thus, collinearity did not exist.

To ensure sufficient power, the minimum sample size was set to 91 when the effect size was medium, the number of predictors was five, the power was 0.8, and the alpha value was 0.05 [39]. When the effect size was small, the minimum sample size was 686. Although the number of subjects in the present study was less than 686, the sample was large enough to minimize variable inflation and improve reliability. Additionally, a cross-validated technique was used to validate the prediction model. In this study, height^2^/impedance, sex, weight, and age were the predictors in the regression model. In the model, the correlation coefficient of height^2^/impedance and FFM_DXA_ was 0.92, and the standardized coefficient β was 0.43; these values explain approximately 43 % of the variance of FFM. The prediction model developed using the G_1_ and G_2_ data was double cross-validated with an RMSE of 2.31 and 2.18 kg, respectively and a bias ± SD of −0.01 ± 3.22 and 0.05 ± 3.12 kg, respectively. The similar results derived by the G_1_ and G_2_ models indicate that the predictive models can accurately predict FFM. The all-subjects predictive model also showed results similar to the G_1_ and G_2_ models in terms of the correlation coefficient, SEE, and CV, thus validating the accuracy of the predictive model. We also randomly assigned subjects into two groups to double cross-validate the regression models. The results were similar to the BF%-matched samples; the regression lines of FFM_BIA_ against FFM_DXA_ developed using randomly assigned data sets demonstrated a similar trend that deviated from an identical line (slope = 0.93 for G_1_ and 0.92 for G_2_; data not shown).

When comparing the results of our predictive model with those of previously published studies on supine-position hand-to-foot BIA measurements, the correlation coefficient and SEE were similar to those of Kotler *et al.* [32] (r^2^ = 0.83, *n* = 256, SEE = approximately 3.0 kg), Sun *et al.* [8] (r^2^ = 0.92, *n* = 1095, RMSE = 2.9 kg), Heitmann *et al.* [40] (r^2^ = 0.90, *n* =139, SEE = 3.6 kg), and Sun *et al.* [8] (r^2^ = 90, n = 734, RMSE = 3.9 kg); however, they were lower than those of Kyle *et al.* [23] (r^2^ = 0.96, *n* = 343, SEE = 1.8 kg) and Deurenberg *et al.* [41] (r^2^ = 0.92, *n* = 661, SEE = 2.6 kg). These results may have been because the correlation coefficient of the predictive value and the measured FFM value tended to be higher in the hand-to-foot model than in the foot-to-foot model [42, 43]; this may be a shortcoming of the foot-to-foot model in assessing FFM.

The Geneva BIA equation published by Kyle *et al.* [23] provides ideal results of a high r^2^ and low SEE (r^2^ = 0.96, LOA = −3.4 to 3.5 kg, and SEE = 1.72 kg); however, their subjects’ BMI range was narrower (17.0–33.8 kg/m^2^) than that of the subjects in our study (15.9–43.1 kg/m^2^). Sun *et al.* [20] and Deurenberg [44] indicated that the estimated results were affected by the level of adiposity. The developed predictive equations in our study overestimated FFM in subgroups with a higher BF% (male, BF%_DXA_ > 30 %; female, BF%_DXA_ > 40 %). When these subjects were excluded and used to develop another model, then the results (*n* = 442; BMI, 15.8–36.9 kg/m^2^; r^2^ = 0.94; SEE = 2.80 kg, LOA = not reported) were comparable with those reported by Kyle *et al.* [23]. Although these results may be appealing, they have limited application. We included subjects with a high BF% to broaden the application range. The average FFM_DXA_ of the subjects in our study was 50.17 ± 11.25 kg, while that in the Geneva BIA equation was 54.0 ± 10.5 kg. Their sample had a smaller SD for FFM_DXA_, indicating that their data tended to be closer to the mean, resulting in a smaller SEE. The standing foot-to-foot impedance measurement may be convenient, but has a significantly smaller FFM correlation than the hand-to-foot impedance measurement [31, 42]. Based on the estimate equation suggested in our study, the LOA may be large; however, we consider it acceptable. This is one of the limitations of the present study. When estimating FFM using our predictive equation in subjects with a high level of adiposity (female, BF%_DXA_ > 40 %; male, BF%_DXA_ > 30 %), the bias ± SD in female and male subjects was 2.0 ± 2.9 and 2.1 ± 3.2 kg, respectively. Although the bias and SD were higher than those in the other leaner subgroups in our study, the results show that our predictive equations performed better for estimating FFM in subjects with a high BF% than did the equation developed by Jakicic *et al.* [45] (r^2^ = 0.66, SEE = 8.8 kg, *n* = 123, and LOA = not reported).

Age has not been included as a predictor in every model in other published studies [23, 36, 41]. Some models excluded age because it only explained limited variance in FFM [23]. However, in our predictive model, age explained approximately 18.0 % of the variance in FFM and was therefore included in the predictive model. Several studies have indicated that the concentration of potassium in fat-free tissue decreases systematically with age [46, 47]. There are important age-related changes in the composition of FFM. The main molecular components of the FFM are water, protein, osseous and nonosseous mineral, and glycogen. The proportion of water, protein, and osseous mineral in the FFM vary systematically with age. Kyle *et al.* [47] examined the accuracy of a predictive model with different age groups. Many studies have reported that the accuracy of BIA estimation is affected by the level of obesity [18–20]. Therefore, this study examined the accuracy of a model for predicting FFM in individuals with different percentages of body fat. The predictive value of FFM using our model was not significantly different from FFM_DXA_ among the subgroups of different BF% values and sexes, and the correlation coefficients were 0.87 (*p* = 0.92) in females and 0.89 (*p* = 0.97) in males. These results indicate that BIA is an accurate method for assessing FFM in individuals with a BF% in the range evaluated in our study. Moreover, standing foot-to-foot BIA can be used as a convenient method to assess the different BF% values in male and female adults. Clinical use of BIA in patients with abnormal hydration cannot be recommended until further validation has proven that a BIA algorithm is accurate in such conditions. The present study focused on the different foot-to-foot BIA BF% values; we did not discuss differences in foot-to-foot BIA FFM estimate measurements based on either regional composition or different body types; these are topics requiring further discussion. Moreover, in patients with body shape abnormalities, very small or large body heights, or relative sitting heights, the use of prediction equations in subjects with an abnormal body build (*e.g.*, acromegaly or amputation) should be interpreted with caution [48]. Many published studies on BIA estimate equations have used impedance as an estimate variable, but the present study applied the impedance variable in the standing foot-to-foot model and found satisfactory results for estimating FFM in a healthy Taiwanese population (BMI = 16–43 kg/m^2^).

## Conclusions

Our FFM predictive model based on standing foot-to-foot BIA can conveniently predict FFM in both male and female healthy Asian subjects with different BF% values.

## Consent

Written informed consent was obtained from the patient for the publication of this report and any accompanying images.

## Abbreviations

BF%: Body fat percentage
H: Height
Z_F-F_: Foot-to-foot impedance
w: Weight
y: Age
s: Sex
Xc: Reactance
R: Resistance
LOA: Limits of agreement
